# Polysaccharides from Basidiocarps of Cultivating Mushroom *Pleurotus ostreatus*: Isolation and Structural Characterization

**DOI:** 10.3390/molecules24152740

**Published:** 2019-07-28

**Authors:** Ekaterina Baeva, Roman Bleha, Ekaterina Lavrova, Leonid Sushytskyi, Jana Čopíková, Ivan Jablonsky, Pavel Klouček, Andriy Synytsya

**Affiliations:** 1 Department of Carbohydrates and Cereals, Faculty of Food and Biochemical Technology, University of Chemistry and Technology in Prague, Technická 5, 166 28 Praha 6 Dejvice, Czech Republic; 2 Department of Gardening, Faculty of Agrobiology, Food and Natural Resources, Czech University of Life Sciences Prague, Kamýcká 129, 165 00 Praha 6 Suchdol, Czech Republic; 3 Department of Crop Production, Faculty of Agrobiology, Food and Natural Resources, Czech University of Life Sciences Prague, Kamýcká 129, 165 00 Praha 6 Suchdol, Czech Republic

**Keywords:** oyster mushrooms, basidiocarps, polysaccharides, fractionation, mannogalactan, glucans

## Abstract

Oyster mushrooms are an interesting source of biologically active glucans and other polysaccharides. This work is devoted to the isolation and structural characterization of polysaccharides from basidiocarps of the cultivated oyster mushroom, *Pleurotus ostreatus*. Five polysaccharidic fractions were obtained by subsequent extraction with cold water, hot water and two subsequent extractions with 1 m sodium hydroxide. Branched partially methoxylated mannogalactan and slightly branched (1→6)-β-d-glucan predominated in cold- and hot-water-soluble fractions, respectively. Alternatively, these polysaccharides were obtained by only hot water extraction and subsequent two-stage chromatographic separation. The alkali-soluble parts originating from the first alkali extraction were then fractionated by dissolution in dimethyl sulfoxide (DMSO). The polysaccharide insoluble in DMSO was identified as linear (1→3)-α-d-glucan, while branched (1→3)(1→6)-β-d-glucans were found to be soluble in DMSO. The second alkaline extract contained the mentioned branched β-d-glucan together with some proteins. Finally, the alkali insoluble part was a cell wall complex of chitin and β-d-glucans.

## 1. Introduction

For millennia, mushrooms have been valued by humankind as an edible and medical resource. Isolation and structural characterization of new compounds, including cell wall polysaccharides, from various mushroom sources is of interest for the search of biologically active agents [1]. The genus *Pleurotus,* including oyster mushrooms, is interesting because these mushrooms can be easily cultivated due to their high adaptability and productivity. Several types of polysaccharides have been isolated from oyster mushrooms. Among them, the most studied is the (1→3)-β-d-glucan named pleuran [2], which has been extracted from fruiting bodies of *Pleurotus ostreatus*, a popular and important specie cultivated commercially worldwide. It has been reported that dietary supplements based on pleuran had hypocholesterolemic effects in animal models [3]. In addition, a number of studies demonstrated that this polysaccharide had many medicinal effects in human organisms including immune modulation and antibacterial activity [4,5,6]. Many other species of genus *Pleurotus* have been used for the isolation and characterization of bioactive glucans, for example *P. eryngii* [7,8], *P. florida* [9,10], *P. ostreatoroseus* [7,11], *P. pulmonarius* [12,13], *P. tuber-regium* [14] and *P. sajor-caju* [15,16,17]. Besides various glucans, water-soluble branched 3-*O*-methylated mannogalactans were also isolated from these mushrooms [18,19]. Several reports are devoted to isolation and fractionation strategy for water-soluble and water-insoluble polysaccharides from basidiocarps of oyster mushrooms [7,20].

The immune modulating activity of cell wall polysaccharides is very important for the medicinal use of oyster mushrooms [21,22]. However, recent studies have shown that the effects of various cell wall components of parasitic fungi on the immune response can be very diverse [23]. Fungal polysaccharides of the inner cell wall (β-glucans, chitin) are very conservative in their structure. These polysaccharides can trigger the strongest innate immune response, and the immune system is able to recognize them [24,25]. On the contrary, polysaccharides or glycoproteins in the outer cell walls (α-glucans, heteropolysaccharides, mannoproteins, etc.) demonstrate a large structural diversity. These molecules are involved in immune avoidance, can mask the internal polysaccharides from immune recognition and cause an anti-inflammatory effect [26]. Consequently, the immune response to a mixture of fungal polysaccharides may differ from that observed with isolated and purified ones [23]. Also, the presence of other compounds, such as proteins, polyphenols and lipids, can affect the biological activity of the fungal cell wall components. Purification and structural characterization of fungal cell wall polysaccharides is thus very important for their further application as selective and effective immune modulators. In the case of oyster mushrooms, it is important to improve the processes of isolation and purification and, thus, to get more purified bioactive polysaccharides.

This work is devoted to subsequent isolation and structural characterization of water- and alkali-soluble polysaccharides from basidiocarps of cultivated oyster mushroom *Pleurotus ostreatus* using improved preparative and analytical approaches.

## 2. Results and Discussion

### 2.1. Yields of Isolations

Yields of isolated products are summarized in Table 1. It is evident that the highest yield of extracted products was in the case of 1st alkaline extraction (**F3**), while the next alkaline extraction led to in order lower yield (**F4**). The yield of cold water extraction (**F1**) was about two times higher than that of subsequent hot water extraction (**F2**). By contrast, the differences in the yields of products **F1′** and **F2′** were not so pronounced. The yield of the dimethyl sulfoxide (DMSO)-soluble part of the 1st alkaline extract (**F3a**) was less pronounced than the yield of the insoluble part (**F3b**). Around a half of the raw material was retained as solid after all extractions (**F5**).

### 2.2. Organic Elemental Composition

Contents of organic elements in the crude fractions are represented in Table 2. High contents of nitrogen in **F1** (3.81%), **F1′** (2.58%) and **F4** (2.01%) as well as sulfur found in these fractions originated from proteins. The protein contents calculated from the nitrogen amounts were 23.81% for **F1**, 16.13% for **F1′** and 12.56% for **F4**; the corresponding values of nitrogen to sulfur (N/S) ratio were 10.6, 9.2 and 13.4. By contrast, fractions **F2** and **F3a,b** contained low amounts of nitrogen (0.2–0.5%); marked amounts of sulfur in **F3a** (0.75%) and **F3b** (0.23%) indicate residues of dimethyl sulfoxide. Nitrogen in the insoluble part F5 (2.22%) originated from both chitin and proteins. The amounts of nitrogen fractions in **F5** originated from proteins (0.92–1.34) and chitin (0.88–1.30) were calculated based on the N/S values mentioned above. According to these values, the contents of proteins and chitin in **F5** were 5.76–8.38% and 12.77–18.85%, respectively, that is in agreement with the data previously reported for insoluble cell wall materials isolated from basidiocarps of *Pleurotus ostreatus* [27].

### 2.3. Glucan Assay

Enzymatic determination of soluble and insoluble glucans in the crude fractions is summarized in Table 3. It was found that glucans are minor in **F1**, so this fraction consists of polysaccharides structurally different from glucans. By contrast, only β-glucans are pronounced in fractions **F2**, **F3** and **F5** (45–60% *w*/*w*). The contents of α-glucans were negligible for all the fractions (less than 1% *m*/*m*). This result is contrary to that, as it is evident from the spectroscopic data (see below), alkali soluble fraction **F3** contained two types of glucans, i.e., linear (1→3)-α-d-glucan and branched (1→3)(1→6)-β-d-glucan. Similarly as it was earlier suggested for glucans in basidiocarps (pilei and stems) of *P. ostreatus* (various strains) and *P. eryngii* [28], the former glucan in the isolated state (as part of **F3**) is possibly not available or only partially available for the amylolytic enzymes used in the kit, so its amount is counted together with β-d-glucan. Therefore, in this case the glucan fractions should be defined as “starch-like α-d-glucan” and “non-starch glucans” as used previously [7,27]. More investigations on (1→3)-α-d-glucan hydrolysis by amylolytic enzymes should be made to clarify this discrepancy.

### 2.4. Monosaccharide Composition

The results of monosaccharide composition analysis are summarized in Table 4. Glucose was found to be prevailing monosaccharide (95–97 mol.%) in the fractions **F2–5**. By contrast, **F1** contained significant amounts of galactose and mannose (molar ratio about 1.6: 1) as well as other sugars including glucose (2.0–10.6 mol.%). The monosaccharide composition confirmed that various types of glucans predominated in **F2–5**, while **F1** contained heteropolysaccharides, mainly mannogalactans. Indeed, water-soluble *O*-2-β-d-manno-*O*-3-methoxy-(1→6)-α-d-galactans, which may also have α-d-galactose [29] or *O*-3-methoxy-α-d-galactose [19,30] as side chains, and are common for various oyster mushrooms [18,31,32], but other water-soluble heteropolysaccharides like *O*-2-β-d-gluco-(1→6)-α-d-galactan [33], *O*-3-α-galacto-(1→6)(1→3)-β-glucan [33] and *O*-2-β-manno- (1→4)-α-gluco-(1→6)-α-galactan [34] were also described. Otherwise, the minor sugars of **F1** could originate from glycoproteins that have been also described for these mushrooms [35,36]. Fraction **F1′** contained significantly less galactose and mannose but more glucose (48.4 mol.%), so this is a mixture of glucans and mannogalactan.

### 2.5. FTIR Spectra

**Figure 1:**
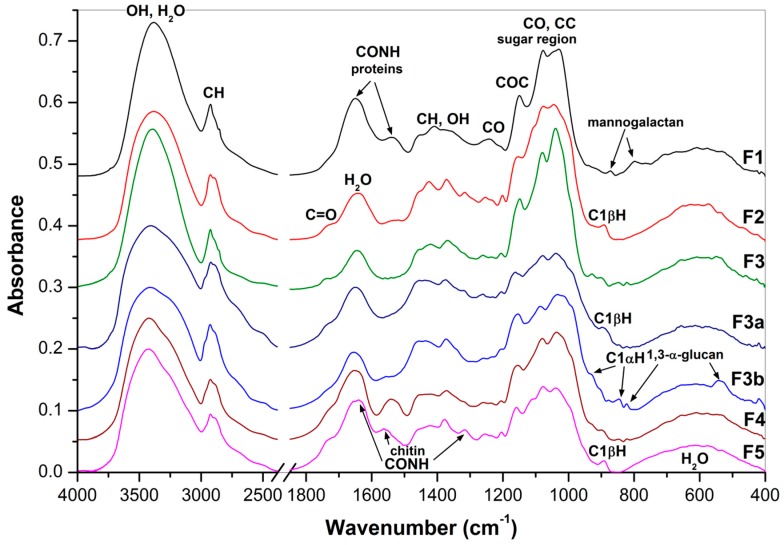
FTIR spectra of the polysaccharidic fractions **F1–3, F3a, F3b, F4** and **F5**.

**Figure 2:**
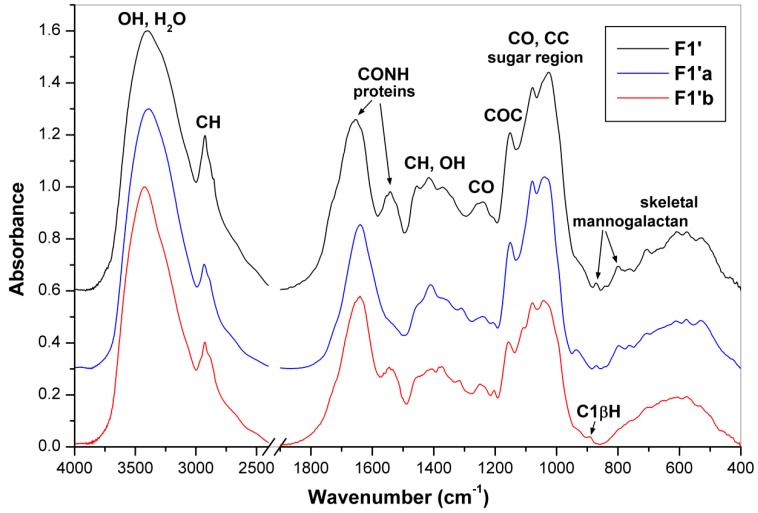
FTIR spectra of the polysaccharidic fractions **F1′**, **F1′a** and **F1′b**.

Fourier transform infrared (FTIR) spectra of the obtained fractions are shown in Figure 1 and Figure 2. The pronounced IR bands were assigned to carbohydrates and other constituents like water, proteins and aromatic compounds. Based on characteristic bands of these compounds it was found that polysaccharides predominated in all the fractions. Bands of proteins at 1653 cm^−1^ (amide I) and 1540 cm^−1^ (amide II) [37] and of mannogalactans at 874 and 800 cm^−1^ assigned to C1H bending and glycosidic bonds of β-d-mannopyranose and α-d-galactopyranose units, respectively [38], were found in the spectra of fractions **F1**, **F1′** and **F1′a**. The latter two bands have been also reported for seed galactomannans [39]. The characteristic bands of β-glucans at 1374, 1318, 1158–1160, 1080, 1038–1040 and 890–894 cm^−1^ [7,40,41] were found in fractions **F1′b**, **F2**, **F4** and **F5**. Comparing the characteristic bands of mannogalactans and β-glucans mentioned above, it is evident that the differences between **F1′a** and **F1′b** were not so marked like those between **F1** and **F2**, so in the former case the separation of these polysaccharides was incomplete. Characteristic glucan bands sensitive to anomeric structures are generally placed at 750–950 cm^−1^, where the bands corresponding to α- or β- anomeric C1H deformation band occur. The band at 890 cm^−1^ is attributed to (1→3)-β-d-glucans, while the bands at 850 and 929 cm^−1^ are characteristic of α-d-glucans, and the band at 822 cm^−1^ specifically to (1→3)-α-d-glucan [7,42]. In addition, two skeletal vibration bands at 543 and 454 cm^−1^ are also attributive to the latter α-d-glucan [42]. The presence of characteristic IR bands of both α- and β-glucans confirmed that fraction **F3** (alkali extract) is a mixture of these two polysaccharides. By contrast, the FTIR spectra of sub-fractions isolated from **F3** confirmed separation of branched (1→3)(1→6)-β-d-glucan (**F3a**) soluble in DMSO from linear (1→3)-α-d-glucan (**F3b**), which was insoluble in this solvent. The former polysaccharide was previously isolated from oyster mushrooms [7,43], whereas the latter one is common in many mushrooms including genus *Pleurotus* [1,2,3,4,5,6,7]. Characteristic IR bands of α-chitin at 1650–1630 cm^−1^ (amide I), 1560 cm^−1^ (amide II), 1382 cm^−1^ (symmetric bending of CH_3_) and 1318 cm^−1^ (amide III) [44] were found in the spectrum of **F5**. However, these bands are of weak intensity like those in the case of chitin-β-d-glucan complexes previously isolated from basidiocarps of *P. ostreatus,* if compared with the corresponding bands of pure chitin or cell wall complexes from other fungal sources [27]. Therefore, the contribution of chitin in **F5** should be relatively low, which is in agreement with the results of the organic elemental analysis (see above).

### 2.6. NMR Spectra

**Figure 3:**
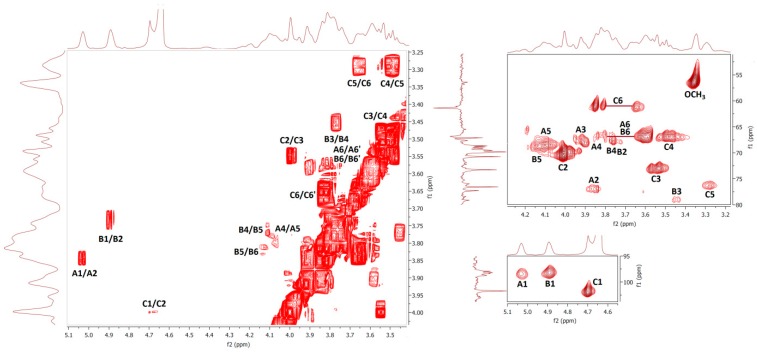
The ^1^H, ^1^H COSY (left) and ^1^H, ^13^C HMQC (right) spectra of fraction **F1**.

**Figure 4:**
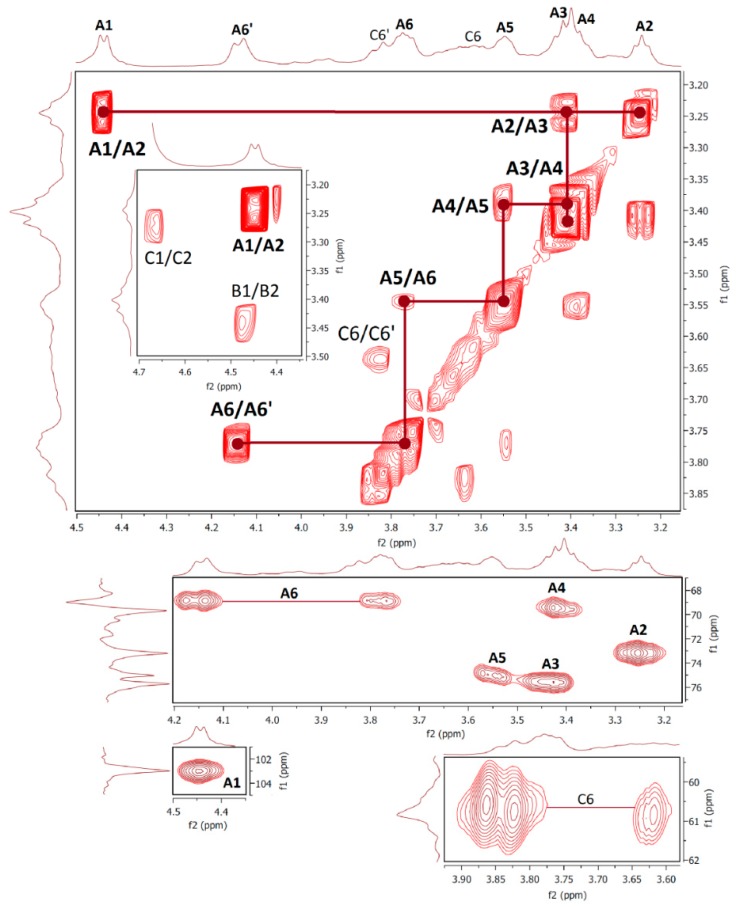
The ^1^H, ^1^H COSY (top) and ^1^H, ^13^C HMQC (bottom) spectra of fraction **F2**.

**Figure 5:**
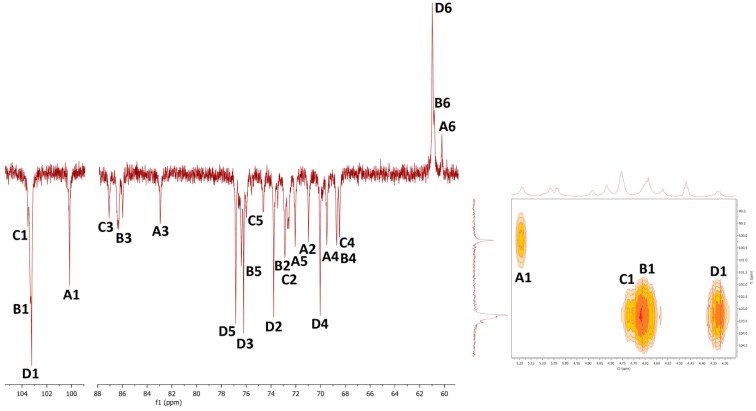
Carbon-13 APT NMR (left) and ^1^H, ^13^C HMQC (right) spectra of sub-fraction **F3a.**

**Figure 6:**
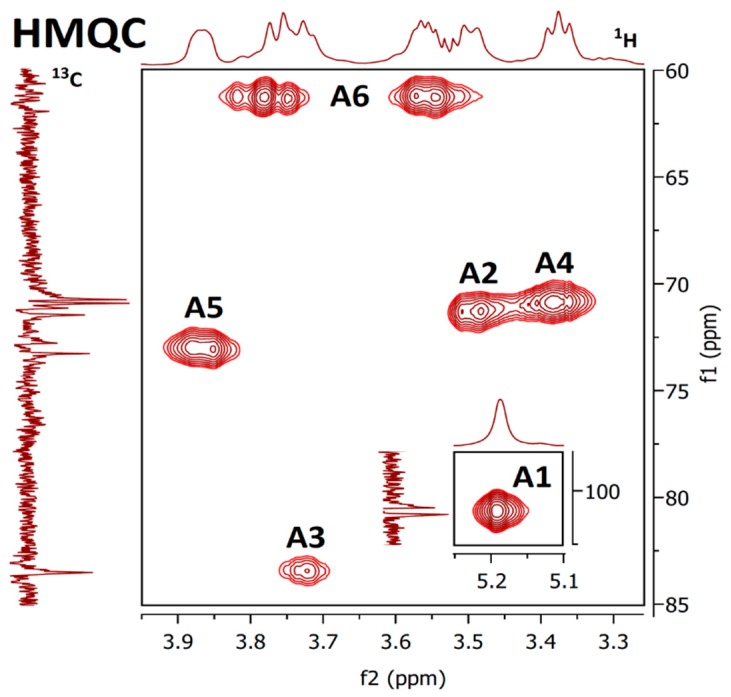
HMQC spectrum of sub-fraction **F3b**.

Results of nuclear magnetic resonance (NMR) spectroscopic study of water- and alkali-soluble fractions **F1**, **F2**, **F3a** and **F3b** are summarized in Table 5; the appropriate spectra are shown in Figure 3, Figure 4, Figure 5 and Figure 6.

NMR spectra of water-soluble fractions **F1** and **F2** were measured in D_2_O at 20 °C and 80 °C, respectively. Correlation NMR spectra of purified **F1** (Figure 3) were assigned to three main sugar residues identified as 1,2,6- and 1,6-linked α-galactoses (A and B units) and terminal β-mannose (C unit). Therefore, the main polysaccharide of this fraction was defined as a highly branched mannogalactan having 1,6-linked α-galactose units in the backbone and terminal β-mannose attached to *O*-2 position of some galactoses [18,31]. In addition, methoxy groups were found to be attached at the *O*-3 position of non-substituted α-galactose in the backbone. By contrast, fraction **F2** was defined as 1,6-linked β-d-glucan because of the pronounced resonances of 1,6-linked β-glucose (A) (Figure 4), but in contrast to linear (1→6)-β-glucan previously isolated from a somatic hybrid of *Pleurotus florida* and *Volvariella volvacea* [43], this polysaccharide was slightly branched at the *O*-3 position, as evident from the resonance signals of two minor units assigned as 1,3,6-linked (B) and terminal (C) β-glucoses.

Fraction **F3** was separated into DMSO-soluble (**F3a**) and DMSO-insoluble (**F3b**) parts by dissolving in this solvent. The NMR spectra of obtained sub-fractions were measured in NaOD/D_2_O and d_6_-DMSO solutions, respectively. In the case of sub-fraction **F3a** (Figure 5), the main residue (D) was identified as terminal β-glucose, the less pronounced residues (A, B and C) as 1,3-linked α-glucose (the same as in case of **F3b**), and 1,3-linked and 1,3,6-linked β-glucose, respectively. By contrast, for sub-fraction **F3b** the main residue (A) was identified as 1,3-linked α-glucose (Figure 6); the signals of β-glucose residues were slightly pronounced. Therefore, linear (1→3)-α-d-glucan predominated in the DMSO insoluble part, while branched (1→3)(1→6)-β-d-glucan did in the DMSO solution, so the treatment of **F3** with DMSO led to separation of these glucans, which is in agreement with FTIR spectra. Similarly, branched β-d-glucan that was insoluble in water, but soluble in DMSO was isolated from sclerotium of *P. tuber-regium* [45].

### 2.7. Preparative Chromatography

**Figure 7:**
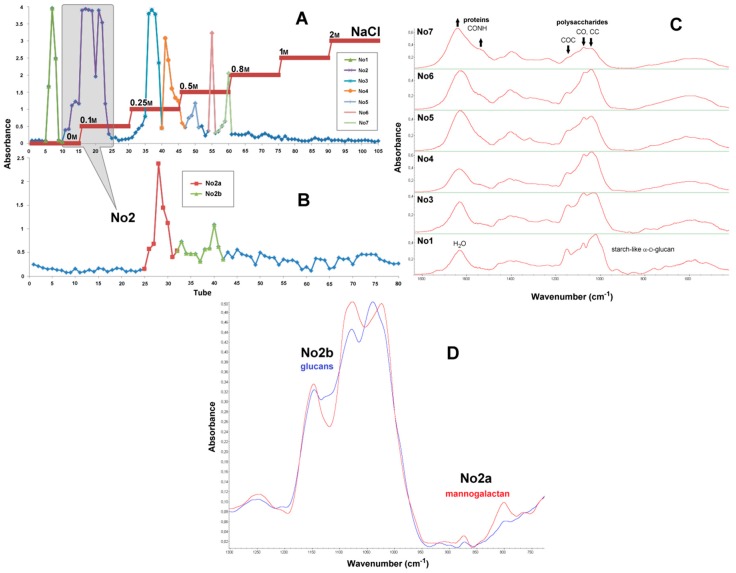
Chromatograms of anion exchange (**A**) and size exclusion (**B**) chromatographic separation of water-soluble polysaccharides from basidiocarps of *P. ostreatus* and FTIR spectra of the obtained sub-fractions (**C,D**).

**Figure 8:**
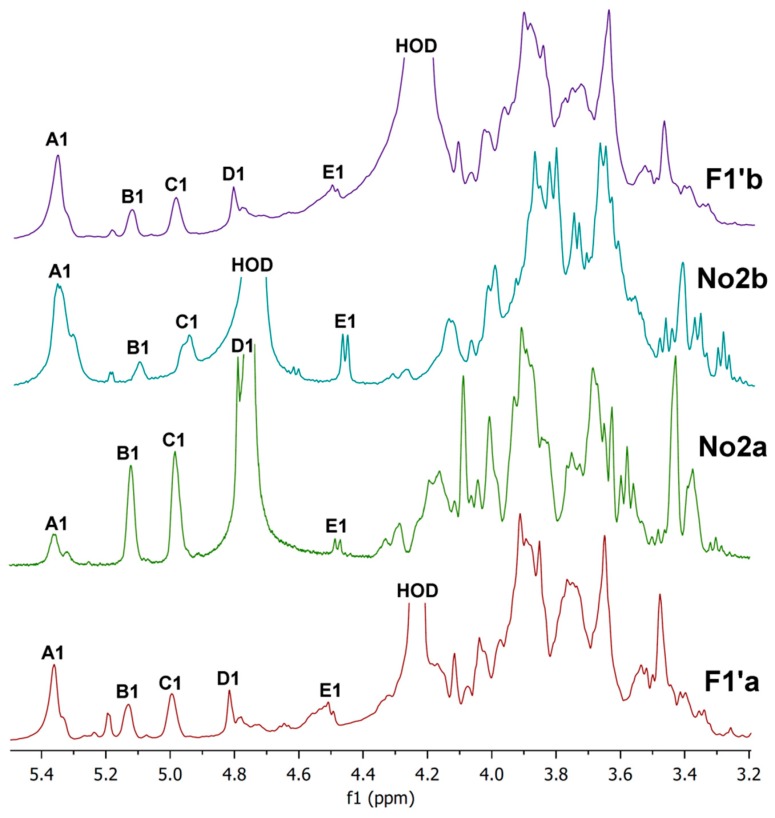
Proton NMR spectra (D_2_O, 20 °C and 80 °C) of cold-water-soluble (**F1′a**) and cold-water-insoluble (**F1′b**) parts and sub-fractions **No2a** and **No2b** of hot water extract **F1′** obtained from basidiocarps of *P. ostreatus*.

Results of chromatographic separation of water-soluble polysaccharides obtained from basidiocarps of *P. ostreatus* by extraction with hot water (**F1′a**, soluble in cold water) are described in Figure 7 (chromatograms, FTIR spectra of sub-fractions) and Figure 8 (^1^H NMR spectra). Firstly, polysaccharides and proteins were separated according to their electrostatic interactions with the cationic stationary phase, yielding seven sub-fractions **No1–7** (Figure 7A). Secondly, the polysaccharides of the main sub-fraction **No2** (tubes 10–25) were separated according to their molecular size on gel column, yielding two sub-fractions **No2a** (tubes 25–32) and **No2b** (tubes 32–42) with increasing molecular weights (Figure 7B). FTIR spectra of the sub-fractions are shown in Figure 7C,D. The spectrum of the first sub-fraction **No1** (tubes 5–10) (Figure 7C) has several bands at 1240, 1154, 1080, 1025, 931, 852, 761, 707, 608, 579 and 529 cm^−1^ typical for starch-like (1→4)(1→6)-α-d-glucan [46]. These bands are absent in the spectra of sub-fractions **No3–7** (tubes 24–61), which also demonstrate pronounced absorbance of carbohydrates at 1200–950 cm^−1^ (the “sugar region”). For sub-fractions **No3–7**, two characteristic bands of the amide vibrations in proteins near 1650 cm^−1^ (amide I) and 1645 cm^−1^ (amide II) [38] in comparison with the “sugar region” showed gradual increases with the elution time, so the protein contribution in these fractions should increase as well. Consequently, the first stage of chromatographic purification led to the removal of a certain amounts of starch-like α-d-glucan and proteins from the main sub-fraction **No2**, which was subjected to subsequent purification on the second column. The FTIR spectra of the resulting sub-fractions **No2a** and **No2b** (Figure 7D) demonstrated significant differences in the envelope of the “sugar region” 1200–950 cm^−1^, which is very sensitive to the composition and structure of polysaccharides, and in the next spectral region of 950–750 cm^−1^ containing bands of skeletal vibrations sensitive to anomeric configuration of sugar units. Indeed, the spectrum of **No2a** has two bands of mannogalactans at 874 and 800 cm^−1^ [39], while the spectrum of **No2b** has no pronounced bands in this region.

Proton NMR spectra of hot water extracts (cold soluble and insoluble), cold-water-soluble (**F1′a**), cold-water-insoluble (**F1′b**) parts and sub-fractions **No2a** and **No2b** of the hot water extract **F1′** are shown in Figure 8. Spectra were measured in D_2_O at 20°C (**No2a**,**b**) and at 80 °C (**F1′a,b**). It is evident from the spectra that both parts of **F1′** contained a mixture of polysaccharides, mainly branched mannogalactan, (1→4)(1→6)-α-d-glucan and (1→6)-β-d-glucan. The five following units were found: 1,4-linked α-glucose (A); 1,2,6-linked α-galactose (B); 1,6-linked α-galactose (C); terminal β-mannose (D); and 1,6-linked β-glucose (E) [18,31,43,47]. By contrast, the ^1^H NMR spectra of the sub-fractions **No2a** and **No2b** confirmed chromatographic separation of the individual polysaccharides. The spectrum of **No2a** contained three intense signals of the anomeric protons B1, C1 and D1 of the mannogalactan units at 5.12, 4.98 and 4.78 ppm, respectively [18,32]. These signals were much more pronounced than the corresponding signals of glucans A1 and E1. Conversely, in the case of **No2b**, two signals of the anomeric protons A1 (α-glucan) and E1 (β-glucan) at 5.36 ppm and 4.47 ppm [43,47], respectively, were the most intense compared to the other anomeric proton signals. Consequently, the branched mannogalactan was successfully separated from glucans by two-step chromatographic purification of the hot water extract **F1′**, as in the case of two-step extractions with cold and hot water yielding **F1** and **F2**, respectively (see above).

## 3. Materials and Methods

### 3.1. Materials

Basidiocarps of cultivated mushrooms *Pleurotus ostreatus* were obtained from mushroom grower Ing. Rudolf Ryzner (Kojátky, Czech Republic). Dimethyl sulfoxide and other chemicals were obtained from Penta Investments (Prague, Czech Republic).

### 3.2. Isolation and Purification Procedures

Polysaccharidic fractions were isolated from lyophilized basidiocarps of *P. ostreatus* by subsequent extractions with cold water (25 °C), hot water (100 °C) and 1m aqueous sodium hydroxide (4 °C) at intense stirring. The homogenized basidiocarps (100 g) were washed with 80% (*w*/*w*) ethanol to remove small molecules (mono- and oligosaccharides, phenolic compounds etc.) and then extracted three times with cold distilled water (600 mL, 25 °C at continuous magnetic stirring) and three times with boiling water (600 mL, 100 °C under reflux) for 7 h per extraction. The solids were separated by centrifugation, and the supernatants were concentrated by vacuum evaporator at 60–65 °C and 40 mBar, lyophilized and merged to give crude cold- and hot-water-soluble fractions, assigned as **F1** and **F2**. For comparison, the isolation of water-soluble polysaccharides was carried out using only extraction with hot water at the same conditions. Obtained extract was lyophilized and then dissolved in 200 mL of distilled water and placed in a refrigerator at 4 °C for 24 h. The resulting precipitate was isolated by centrifugation, washed with 96% ethanol, dried on a glass clock and labeled as **F2′** (insoluble in cold water). The solution was concentrated, lyophilized and assigned as **F1′** (soluble in cold water). The crude products were rigorously washed with 0.4 m hydrochloric acid in 80% (*w*/*w*) ethanol to remove colored substances and other small molecules, then with 80% (*w*/*w*) ethanol without acid until pH 7 and dried in air. The insoluble parts were then used for two subsequent extractions with 1 m aqueous solution of sodium hydroxide (600 mL per each extraction) containing 0.05% of sodium borohydride to avoid oxidation at 4 °C for 12 h per each extraction. The solids were separated by centrifugation, and polysaccharides were precipitated from the supernatant by an excess of ethanol (3:1 *v*/*v*), neutralized by washing with 0.4 m hydrochloric acid in 80% (*w*/*w*) ethanol and then with 80% (*w*/*w*) ethanol without acid until pH 7 and dried in air. Two alkali-soluble fractions were obtained separately and assigned as **F3** and **F4**. The solids remaining after all extractions were neutralized by the same manner and then dried in air, yielding the insoluble fraction **F5**. Soluble polysaccharides were extracted from dried fraction **F3** (1st alkali extraction) with dimethyl sulfoxide (DMSO, 600 mL, 25 °C) at continuous magnetic stirring for 4 h. The extract was separated from pellets by centrifugation. Polysaccharides were precipitated from the extract with an excess of ethanol (3:1 *v*/*v*) yielding DMSO soluble sub-fraction (**F3a**), and the pellet was washed with ethanol to remove reminder solvent and dried on air yielding DMSO insoluble sub-fraction (**F3b**).

Water-soluble polysaccharides were separated and purified using anionic exchange and size exclusion preparative chromatography. Weighted amount of fraction **F1′** (200 mg) was completely dissolved in 1 mL of distilled water at 98°C, cooled and filtrated with PVDF 0.22µm syringe filter. The solution was applied to BenchMark 15 mm column (43 cm × 1.5 cm) (Omnifit, USA) packed with DEAE Sepharose Fast Flow gel (GE Healthcare Life Sciences, Chicago, IL, USA) equilibrated with distilled water. The sub-fractions were eluted by Gilson FC 203B fraction collector (Gilson Inc., Middleton, WI, USA) into 105 tubes with distilled water followed by stepwise gradient of sodium chloride solution (0.1 m, 0.25 m, 0.5 m, 0.8 m, 1, 2 m), with flow rate of 1 mL/min (2.5 min/tube). The phenol-sulfuric acid assay on microplates was then performed to estimate total sugars in each tube from both columns by photometry at 490 nm [48]. The sub-fractions of interest designed as **No1-7** were collected, dialyzed with cut-off membranes for 1 kDa against distilled water for 48 hrs, concentrated and stored. Concentrated sub-fraction **No2** from tubes 10-26 after anion-exchange chromatography was filtrated with PVDF 0.22 µm syringe filter and applied in volume of 1 mL to K16/100 column (93 cm × 1.5 cm) filled with Sephadex G-75 superfine gel (GE Healthcare Life Sciences, Chicago, IL, USA), equilibrated with 0.1 m sodium chloride solution and eluted with the same solution as mobile phase. The sub-fractions were collected in 80 tubes (1.2 mL per a tube) with flow rate of 0.15 mL/min (8 min/tube) and analyzed for total sugar concentration (see above). The chromatograms were processed using Gen5 TS 2.06 (Biotek, Winooski, VT, USA) software and exported in ASCII format to Microsoft Excel 2010 for preparation of graphs. Six sub-fractions from the 1st column (**No1,3-7**) and two sub-fractions from the 2nd column (**No2a,b**) were defined, collected, dialyzed with cut-off membranes for 1kDa against distilled water for 48 h, and lyophilized.

### 3.3. Glucan Assay

The analytical set “MUSHROOM and YEAST Β-GLUCAN” K-YBGL 07/11 (Megazyme International, Ireland) was used for determination of total, α- and β-glucans [7,28]. The assay is based on the difference between glucose contents after the total acidic hydrolysis of glucans and specific enzymatic hydrolysis of starch-like α-glucans. The polysaccharide fractions were solubilized in concentrated (37%; 10 n) hydrochloric acid and then hydrolyzed by 1.3 m hydrochloric acid at 100 °C for 2 h; total hydrolysis was completed by incubation with a mixture of *exo*-1,3-β-glucanase and β-glucosidase. The starch-like α-glucans were solubilized in 2 m potassium hydroxide, and the mixture was neutralized with an excess of 1.2 m sodium acetate buffer (pH 3.8); dissolved α-glucans were then hydrolyzed by amyloglucosidase. The content of β-glucans (or non-starch) glucans was calculated as the difference between glucose contents after total acidic hydrolysis of glucans and specific enzymatic hydrolysis of α-glucans. The glucan contents in the isolated fractions were represented in dry matter.

### 3.4. Organic Elemental Analysis

Organic elementary analysis (C, H, N and S) was made on Elementar vario EL III (Elementar, Germany). The accuracy of the method is determined for simultaneous analysis of 5 mg of 4-amino-benzene sulfonic acid in the CHNS module to <0.1% abs. for each element. The results include all combustible sulfur, both organic and inorganic, as well as all combustible carbon, organically and inorganically bound. The hydrogen content is affected by the moisture of the sample. The amount of residual proteins (% *m*/*m*) in the soluble fractions was calculated as the *N* × 6.25, based on the estimation that the average nitrogen content of proteins in fungal materials is around 16% [49,50]. The amount of chitin in the insoluble part was calculated as *N* × *M*_aGlcNAc_/*M*_N_, where *M*_aGlcNAc_ and *M*_N_ are molecular/atomic masses of anhydro-*N*-acetyl-glucosamine unit (203.21) and nitrogen (14), respectively.

### 3.5. Monosaccharide Composition Analysis

Neutral sugars were determined as alditol acetates by gas chromatography [51]. The samples (1–2 mg) were solubilized in 72% sulfuric acid and the hydrolysis carried out at ambient temperature for 3 h with occasional stirring. Distilled water (2.2 mL) was added and the hydrolysis continued for further 2.5 h at 100 °C and then ended by cooling down in an ice bath. The internal standard of 2-deoxy-d-glucose with a concentration of 1 mg/mL was added to the remaining solution. The volume of 1 mL was transferred and neutralized with 25% ammonium hydroxide. This solution was reduced with 15% sodium borohydride in 3 m ammonium hydroxide, incubated for 1 h at 30 °C and 2 × 50 µl of glacial acetic acid added after cooling in an ice bath. In an ice bath, 450 µl of 1-methylimidazole and 3 mL of acetic anhydride were added and the solution was further incubated for 30 min at 30 °C. The solution was returned into the ice bath where 3 mL distilled water and 2.5 mL dichloromethane were added. To effectively extract the alditol acetates, the solution was vigorously stirred, centrifuged and the aqueous phase removed under vacuum. Three milliliters distilled water and 2.5 mL dichloromethane were added and again, as previously described, the solution was vigorously stirred, centrifuged and the aqueous phase removed under vacuum. The resulting solution of the alditol acetates in dichloromethane was twice washed with distilled water, stirred, centrifuged and the aqueous phase removed under vacuum. In the evaporative concentrator Termovap TV10+ (Chromservis, Prague, Czech Republic), the dichloromethane was evaporated. The alditol acetates were twice washed with 1 mL of anhydrous acetone and then evaporated and stored in an anhydrous ambient. These alditol acetates obtained by the reduction and acetylation of monosaccharide were dissolved in 50 µl of anhydrous acetone and then injected to analyze for monosaccharides in GC-FID Shimadzu GC 2010 (Shimadzu, Japan), capillary column DB-225 (30 m length, 0.25 mm internal diameter, 0.15 µm film thickness). The temperatures of injector and detector were, respectively, 220 ˚C and 230 ˚C. The oven temperature program was the following: 200 ˚C for 1 min then rose to 220 ˚C (40 ˚C/min), temperature 220 ˚C for 7 min, then rose to 230 ˚C (20 ˚C/min) until, reaching the final temperature 230 ˚C for 1 min, in a total of 9 min. The hydrolysis of all samples was performed in duplicate.

### 3.6. Spectroscopic Methods

FTIR spectra of polysaccharide fractions were recorded in KBr tablets on a Nicolet 6700 FTIR spectrometer (Thermo Fisher Scientific, MA, USA) in a spectral range from 4000 to 400 cm^−1^ with a resolution of 2 cm^−1^ and 64 scans. The tablets were prepared using hand press (PIKE Technologies, Fitchburg, WI, USA). The spectra were smoothed and baseline corrected using Omnic 8.0 software (Thermo Fisher Scientific, Waltham, MA, USA) and then exported in ASCII format to Origin 6.0 (OriginLab, Northampton, MA, USA) software for preparation of graphs.

Proton NMR and ^13^C APT NMR spectra of the soluble fractions were recorded on a Bruker Avance III 500 MHz (Bruker, Billerica, MA, USA) in the appropriate deuterated solvents at 20–80 °C. The spectra of water-soluble fractions were recorded in deuterium oxide solutions; the corresponding spectra of alkali-soluble fractions were recorded in NaOD/D_2_O (the DMSO-insoluble part) and deuterated dimethylsulphoxide (the DMSO-soluble part). Correlation ^1^H, ^1^H COSY and ^1^H, ^13^C HMQC NMR experiments were used for signal assignment. The 1D and 2D NMR spectra were processed using Mnova 10.0 (Mestrelab Research S.L., Spain) software.

## 4. Conclusions

Several polysaccharidic fractions were isolated from the basidiocarps of the cultivating medicinal mushroom *Pleurotus ostreatus*. The composition and structure of these fractions were defined based on obtained analytical data. It was found that glucans predominated in all the fractions except the cold water extract, which contained branched mannogalactan together with some proteins. However, the structure of these glucans was quite different dependent on the isolation medium. The main polysaccharide of the hot water extract was (1→6)-β-d-glucan, which may be slightly branched at the *O*-3 position. Mannogalactans are known to be polysaccharides common in oyster mushrooms [18]. By contrast, the β-d-glucans of these mushrooms commonly have a backbone of 1,3-linked glucose units [11,15] rather than 1,6-linked, and (1→3)-linear β-d-glucan was described as well [17]. An immune active linear (1→6)-β-d-glucan of similar structure has been isolated from a somatic hybrid of *Pleurotus florida* and *Volvariella volvacea* [43], and this polysaccharide was not reported to be a constituent of any of the parent species. The isolation procedure presented here and based on the combination of cold and hot water extractions permits the separation of water-soluble polysaccharides, i.e., mannogalactans and (1→6)-β-d-glucans, effectively. Alternatively, only hot water extraction can be carried out to obtain a mixture of these polysaccharides, and finally, as a result of two-stage chromatographic separation, purified individual polysaccharides can be obtained.

First alkali extract contained a mixture of linear (1→3)-α-d-glucan and branched (1→3)(1→6)-β-d-glucan. These polysaccharides were then successfully separated by further extraction with dimethyl sulfoxide, because α-d-glucan was insoluble and β-d-glucan was soluble in this solvent. This extraction step thus can be used instead of the treatment of extracts with a toxic phenolic reagent, which has been used in our previous work [7]. The second alkali extraction yielded a mixture of the same glucans, but together with a significant amount of proteins. Finally, the insoluble part was defined as a complex of (1→3)(1→6)-β-d-glucan with chitin, which is typical for mushroom cell walls [7]. Therefore, the combination of cold and hot water extractions as well as additional extraction with dimethyl sulfoxide led to the separation of the individual water- and alkali-soluble polysaccharides. Obtained fractions will be additionally purified and applied for screening of biological activities.

## Figures and Tables

**Table 1:** Yields of polysaccharidic fractions obtained from basidiocarps of *P. ostreatus*.

Fraction	Description	Yield (% *w*/*w*)	Main Component(s)
**F1**	cold water extract	6.14	mannogalactan + proteins
**F2**	hot water extract	3.40	(1→6)-β-d-glucan
**F1′**	hot water extract	10.32	mannogalactan + glucans + proteins
**F1′a**	cold-water-soluble part	5.83	mannogalactan + glucans + proteins
**F1′b**	cold-water-insoluble part	4.27	mannogalactan + glucans + proteins
**F3**	1st alkaline extract	15.63	(1→3)-α-d-glucan + (1→3)(1→6)-β-d-glucan
**F3a**	DMSO-soluble part	1.80	(1→3)(1→6)-β-d-glucan
**F3b**	DMSO-insoluble part	11.24	(1→3)-α-d-glucan
**F4**	2nd alkaline extract	1.54	(1→3)(1→6)-β-d-glucan + proteins
**F5**	insoluble part	50.86	(1→3)(1→6)-β-d-glucan + chitin

**Table 2:** Elemental composition of the crude polysaccharidic fractions.

Fraction	Content (% *w*/*w*)			
	N	C	H	S
**F1**	3.81	41.11	6.68	0.36
**F1′**	2.58	37.53	6.12	0.28
**F2**	0.51	39.72	6.10	0.09
**F3a**	0.39	40.75	6.78	0.75
**F3b**	0.19	41.83	6.87	0.23
**F4**	2.01	41.93	6.82	0.15
**F5**	2.22	40.47	6.74	0.10

**Table 3:** Contents of glucans in the polysaccharidic fractions.

Content (% *m*/*m* of Dry Matter)	Fraction			
	F1	F2	F3	F5
total glucans	6.9	60.27	55.28	45.51
α-glucans	0.80	0.37	0.07 *	0.01
β-glucans	6.14	59.90	55.21^†^	45.49

*starch-like α-glucans; ^†^ non-starch glucans.

**Table 4:** Molar ratio (%) of monosaccharides in the polysaccharidic fractions.

Fraction	Molar Ratio (%)						
	Glc	Fuc	Rha	Gal	Xyl	Man	Ara
**F1**	10.6	2.0	3.5	45.6	4.6	28.3	5.4
**F1‘**	48.4	1.9	2.7	17.8	4.4	18.8	6.0
**F2**	97.1	0.1	0.2	0.9	0.3	1.2	0.2
**F3**	96.0	0.3	0.2	0.6	0.1	0.9	1.9
**F5**	94.9	0.6	1.2	0.6	1.1	0.8	0.8

**Table 5:** Proton and ^13^C resonance signal assignments for the polysaccharidic fractions.

**Fraction F1**								
**Unit**	**H1/C1**	**H2/C2**	**H3/C3**	**H4/C4**	**H5/C5**	**H6/C6**	**OCH_3_**	**Assignment**
**A**	5.04	3.84	3.91	3.80	4.09	3.81; 3.59		1,2,6-β-Gal*p*
	98.12	77.02	68.57	66.84	68.91	66.76		
**B**	4.89	3.73	3.44	3.77	4.12	3.81; 3.59	3.35	1,6-β-Gal*p*3Me
	98.27	68.41	78.87	66.90	68.91	66.74	56.55	
**C**	4.72	4.01	3.56	3.48	3.29	3.82; 3.63		t-α-Man*p*
	101.76	70.53	73.06	66.89	76.28	61.20		
Fraction **F2**								
**Unit**	**H1/C1**	**H2/C2**	**H3/C3**	**H4/C4**	**H5/C5**	**H6/C6**		**Assignment**
**A**	4.44	3.24	3.41	3.38	3.55	3.77		1,6-β-Glc*p*
	103.07	73.17	75.50	69.42	75.00	68.96		
**B**	4.46	3.47	3.68	3.48	3.58	3.77		1,3,6-β-Glc*p*
		72.86	84.55	68.85	74.87	68.96		
**C**	4.66	3.27	3.45	3.32	3.37	3.64		t-β-Glc*p*
		73.17	75.70	69.66		60.84		
Fraction **F3a**								
**Unit**	**H1/C1**	**H2/C2**	**H3/C3**	**H4/C4**	**H5/C5**	**H6/C6**		**Assignment**
**B**	4.74	3.60	3.82	3.55	3.45	3.98; 3.73		1,3-β-Glc*p*
	103.25	73.12	86.61	68.95	76.50	61.10		
**C**	4.75	3.61	3.86	3.59	3.68	4.23; 3.85		1,3,6-β-Glc*p*
	103.35	72.89	86.25	68.95	76.22	68.66		
**D**	4.53	3.31	3.46	3.38	3.41	3.98; 3.73		t-β-Glc*p*
	103.57	73.93	76.45	70.30	77.12	61.23		
Fractions **F3b**								
**Unit**	**H1/C1**	**H2/C2**	**H3/C3**	**H4/C4**	**H5/C5**	**H6/C6**		**Assignment**
**A**	5.19	3.50	3.72	3.38	3.87	3.78; 3.56		1,3-α-Glc*p*
	100.92	71.25	83.45	70.83	73.07	61.22		

## References

[1] Wasser S.P. (2002). Medicinal mushrooms as a source of antitumor and immunomodulating polysaccharides. Appl. Microbiol. Biotechnol..

[2] Karácsonyi Š., Kuniak Ľ. (1994). Polysaccharides of *Pleurotus ostreatus*: Isolation and structure of pleuran, an alkali-insoluble β-D-glucan. Carbohydr. Polym..

[3] Bobek P., Ozdín Ĺ., Kuniak Ĺ. (1997). Effect of oyster mushroom and isolated β-glucan on lipid peroxidation and on the activities of antioxidative enzymes in rats fed the cholesterol diet. J. Nutr. Biochem..

[4] Jesenak M., Majtan J., Rennerova Z., Kyselovic J., Banovcin P., Hrubisko M. (2013). Immunomodulatory effect of pleuran (β-glucan from *Pleurotus ostreatus*) in children with recurrent respiratory tract infections. Int. Immunopharmacol..

[5] Majtan J. (2012). Pleuran (β-glucan from *Pleurotus ostreatus*): An effective nutritional supplement against upper respiratory tract infections?. Med. Sport Sci..

[6] Bergendiova K., Tibenska E., Majtan J. (2011). Pleuran (β-glucan from *Pleurotus ostreatus*) supplementation, cellular immune response and respiratory tract infections in athletes. Eur. J. Appl. Physiol..

[7] Synytsya A., Míčková K., Synytsya A., Jablonský I., Spěváček J., Erban V., Kováříková E., Čopíková J. (2009). Glucans from fruit bodies of cultivated mushrooms *Pleurotus ostreatus* and *Pleurotus eryngii*: Structure and potential prebiotic activity. Carbohydr. Polym..

[8] Akyuz M., Kirbag S. (2009). Antimicrobial activity of *Pleurotus eryngii* var. *ferulae* grown on various agro-wastes. Eur. Asian J. BioSci..

[9] Dey B., Bhunia S.K., Maity K.K., Patra S., Mandal S., Maiti S., Maiti T.K., Sikdar S.R., Islam S.S. (2012). Glucans of *Pleurotus florida* blue variant: Isolation, purification, characterization and immunological studies. Int. J. Biol. Macromol..

[10] Santos-Neves J.C., Pereira M.I., Carbonero E.R., Gracher A.H.P., Alquini G., Gorin P.A., Sasssaki G.L., Iacomini M. (2008). A novel branched αβ-glucan isolated from the basidiocarps of the edible mushroom *Pleurotus florida*. Carbohydr. Polym..

[11] Carbonero E.R., Gracher A.H.P., Smiderle F.R., Rosado F.R., Sassaki G.L., Gorin P.A., Iacomini M. (2006). A β-glucan from the fruit bodies of edible mushrooms *Pleurotus eryngii* and *Pleurotus ostreatoroseus*. Carbohydr. Polym..

[12] Smiderle F.R., Olsen L.M., Carbonero E.R., Baggio C.H., Freitas C.S., Marcon R., Santos A.R.S., Gorin P.A., Iacomini M. (2008). Anti-inflammatory and analgesic properties in a rodent model of a (1→3),(1→6)-linked β-glucan isolated from *Pleurotus pulmonarius*. Eur. J. Pharmacol..

[13] Lavi I., Levinson D., Peri I., Tekoah Y., Hadar Y., Schwartz B. (2010). Chemical characterization, antiproliferative and antiadhesive properties of polysaccharides extracted from *Pleurotus pulmonarius* mycelium and fruiting bodies. Appl. Microbiol. Biotechnol..

[14] Wong S.M., Wong K.K., Chiu L.C.M., Cheung P.C.K. (2007). Non-starch polysaccharides from different developmental stages of *Pleurotus tuber-regium* inhibited the growth of human acute promyelocytic leukemia HL-60 cells by cell-cycle arrest and/or apoptotic induction. Carbohydr. Polym..

[15] Carbonero E.R., Ruthes A.C., Freitas C.S., Utrilla P., Gálvez J., da Silva E.V., Sassaki G.L., Gorin P.A.J., Iacomini M. (2012). Chemical and biological properties of a highly branched β-glucan from edible mushroom *Pleurotus sajor-caju*. Carbohydr. Polym..

[16] Pramanik M., Chakraborty I., Mondal S., Islam S.S. (2007). Structural analysis of a water-soluble glucan (Fr. I) of an edible mushroom, *Pleurotus sajor-caju*. Carbohydr. Res..

[17] Silveira M.L., Smiderle F.R., Moraes C.P., Borato D.G., Baggio C.H., Ruthes A.C., Wisbeck E., Sassaki G.L., Cipriani T.R., Furlan S.A. (2014). Structural characterization and anti-inflammatory activity of a linear β-d-glucan isolated from *Pleurotus sajor-caju*. Carbohydr. Polym..

[18] Smiderle F.R., Olsen L.M., Carbonero E.R., Marcon R., Baggio C.H., Freitas C.S., Santos A.R.S., Torri G., Gorin P.A.J., Iacomini M. (2008). A 3-*O*-methylated mannogalactan from *Pleurotus pulmonarius*: Structure and antinociceptive effect. Phytochemistry.

[19] Zhang A.Q., Xu M., Fu L., Sun P.L. (2013). Structural elucidation of a novel mannogalactan isolated from the fruiting bodies of *Pleurotus geesteranus*. Carbohydr. Polym..

[20] Palacios I., García-Lafuente A., Guillamón E., Villares A. (2012). Novel isolation of water-soluble polysaccharides from the fruiting bodies of *Pleurotus ostreatus* mushrooms. Carbohydr. Res..

[21] Corrêa R.C.G., Brugnari T., Bracht A., Peralta R.M., Ferreira I.C. (2016). Biotechnological, nutritional and therapeutic uses of *Pleurotus* spp. (Oyster mushroom) related with its chemical composition: A review on the past decade findings. Trends Food Sci. Technol..

[22] Patel Y., Naraian R., Singh V.K. (2012). Medicinal properties of Pleurotus species (oyster mushroom): A review. World J. Fungal Plant Biol..

[23] Snarr B., Qureshi S., Sheppard D. (2017). Immune recognition of fungal polysaccharides. J. Fungi.

[24] Goodridge H.S., Wolf A.J., Underhill D.M. (2009). β-Glucan recognition by the innate immune system. Immunol. Rev..

[25] Bueter C.L., Specht C.A., Levitz S.M. (2013). Innate sensing of chitin and chitosan. PLoS Pathog..

[26] Rappleye C.A., Eissenberg L.G., Goldman W.E. (2007). *Histoplasma capsulatum* α-(1, 3)-glucan blocks innate immune recognition by the β-glucan receptor. Proc. Natl. Acad. Sci. USA.

[27] Gomba G.K., Synytsya A., Švecová P., Coimbra M.A., Čopíková J. (2015). Distinction of fungal polysaccharides by N/C ratio and mid infrared spectroscopy. Int. J. Biol. Macromol..

[28] Synytsya A., Míčková K., Jablonsky I., Sluková M., Copikova J. (2008). Mushrooms of genus *Pleurotus* as a source of dietary fibres and glucans for food supplements. Czech J. Food Sci..

[29] Rout D., Mondal S., Chakraborty I., Islam S.S. (2006). The structure of a polysaccharide from Fraction-II of an edible mushroom, *Pleurotus florida*. Carbohydr. Res..

[30] Zhang A.Q., Zhang Y., Yang J.H., Sun P.L. (2013). Structural elucidation of a novel heteropolysaccharide from the fruiting bodies of *Pleurotus eryngii*. Carbohydr. Polym..

[31] Rosado F.R., Carbonero E.R., Claudino R.F., Tischer C.A., Kemmelmeier C., Iacomini M. (2003). The presence of partially 3-*O*-methylated mannogalactan from the fruit bodies of edible basidiomycetes *Pleurotus ostreatus* ‘florida’Berk. and *Pleurotus ostreatoroseus* Sing. FEMS Microbiol. Lett..

[32] Sun Y., Liu J. (2009). Purification, structure and immunobiological activity of a water-soluble polysaccharide from the fruiting body of *Pleurotus ostreatus*. Bioresour. Technol..

[33] Maity K.K., Patra S., Dey B., Bhunia S.K., Mandal S., Das D., Majumdar D.K., Maiti S., Maiti T.K., Islam S.S. (2011). A heteropolysaccharide from aqueous extract of an edible mushroom, *Pleurotus ostreatus* cultivar: Structural and biological studies. Carbohydr. Res..

[34] Pramanik M., Mondal S., Chakraborty I., Rout D., Islam S.S. (2005). Structural investigation of a polysaccharide (Fr. II) isolated from the aqueous extract of an edible mushroom *Pleurotus sajor-caju*. Carbohydr. Res..

[35] Chen J.N., Wang Y.T., Wu J.S.B. (2009). A glycoprotein extracted from golden oyster mushroom *Pleurotus citrinopileatus* exhibiting growth inhibitory effect against U937 leukemia cells. J. Agric. Food Chem..

[36] Chen J.N., de Mejia E.G., Wu J.S.B. (2011). Inhibitory effect of a glycoprotein isolated from golden oyster mushroom (*Pleurotus citrinopileatus*) on the lipopolysaccharide-induced inflammatory reaction in RAW 264.7 macrophage. J. Agric. Food Chem..

[37] Kong J., Yu S. (2007). Fourier transform infrared spectroscopic analysis of protein secondary structures. Acta Biochim. Biophys. Sinica.

[38] Figueiro S.D., Góes J.C., Moreira R.A., Sombra A.S.B. (2004). On the physico-chemical and dielectric properties of glutaraldehyde crosslinked galactomannan–collagen films. Carbohydr. Polym..

[39] Cerqueira M.A., Souza B.W., Simões J., Teixeira J.A., Domingues M.R.M., Coimbra M.A., Vicente A.A. (2011). Structural and thermal characterization of galactomannans from non-conventional sources. Carbohydr. Polym..

[40] Gutiérrez A., Prieto A., Martínez A.T. (1996). Structural characterization of extracellular polysaccharides produced by fungi from the genus *Pleurotus*. Carbohydr. Res..

[41] Šandula J., Kogan G., Kačuráková M., Machová E. (1999). Microbial (1→3)-β-d-glucans, their preparation, physico-chemical characterization and immunomodulatory activity. Carbohydr. Res..

[42] Wang T., Deng L., Li S., Tan T. (2007). Structural characterization of a water insoluble α-(1→3)-d-glucan isolated from the *Penicillium chrysogenum*. Carbohydr. Polym..

[43] Das D., Mondal S., Roy S.K., Maiti D., Bhunia B., Maiti T.K., Sikdar S.R., Islam S.S. (2010). A (1→6)-β-glucan from a somatic hybrid of *Pleurotus florida* and *Volvariella volvacea*: Isolation, characterization, and study of immunoenhancing properties. Carbohydr. Res..

[44] Cárdenas G., Cabrera G., Taboada E., Miranda S.P. (2004). Chitin characterization by SEM, FTIR, XRD, and ^13^C cross polarization/mass angle spinning NMR. J. Appl. Polymer Sci..

[45] Zhang L., Zhang M., Dong J., Guo J., Song Y., Cheung P.C.K. (2001). Chemical structure and chain conformation of the water-insoluble glucan isolated from *Pleurotus tuber-regium*. Biopolym. Orig. Res. Biomol..

[46] Fan D., Ma W., Wang L., Huang J., Zhao J., Zhang H., Chen W. (2012). Determination of structural changes in microwaved rice starch using Fourier transform infrared and Raman spectroscopy. Starch-Stärke.

[47] Nilsson G.S., Gorton L., Bergquist K.E., Nilsson U. (1996). Determination of the degree of branching in normal and amylopectin type potato starch with ^1^H-NMR spectroscopy improved resolution and two-dimensional spectroscopy. Starch-Stärke.

[48] Masuko T., Minami A., Iwasaki N., Majima T., Nishimura S.I., Lee Y.C. (2005). Carbohydrate analysis by a phenol-sulfuric acid method in microplate format. Anal. Biochem..

[49] Schiavone M., Vax A., Formosa C., Martin-Yken H., Dague E., François J.M. (2014). A combined chemical and enzymatic method to determine quantitatively the polysaccharide components in the cell wall of yeasts. FEMS Yeast Res..

[50] Ivshin V.P., Artamonova S.D., Ivshina T.N., Sharnina F.F. (2007). Methods for isolation of chitin-glucan complexes from higher fungi native biomass. Polymer Sci. Ser. B.

[51] Passos C.P., Coimbra M.A. (2013). Microwave superheated water extraction of polysaccharides from spent coffee grounds. Carbohydr. Polym..

